# Building capacity for evidence-informed decision making in Canadian public health

**DOI:** 10.1186/1748-5908-10-S1-A21

**Published:** 2015-08-20

**Authors:** Maureen Dobbins, Robyn Traynor, Lori Greco

**Affiliations:** 1 Health Evidence, School of Nursing, Faculty of Health Sciences, McMaster University, Hamilton, ON L8P 0A1, Ontario, Canada

## Objective

Our research team partnered with three Canadian public health departments to study the impact of tailored knowledge translation and exchange (KTE) strategies on evidence-informed decision making (EIDM). We aimed to enhance public health EIDM knowledge, skills and behaviour and further facilitate organizational contexts conducive to EIDM.

## Methods

We used case study methodology and tailored the intervention and analysis to each unique case (i.e. health department). An experienced Knowledge Broker supported each case through a variety of strategies: large-group training with front-line staff; one-on-one consultation with specialists, guiding them through a structured EIDM process; and advice to management on organizational policies and procedures. Data were collected prior to, during, and following the intervention via an online survey (demographic information, self-reported EIDM behaviours, and social networks) and in-person assessment (EIDM knowledge and skills).

## Results

Results across the three cases revealed a significant increase in EIDM knowledge and skills at follow-up, among those who worked closely with the Knowledge Broker (2.8 points out of a possible 36 points, (95% CI 2.0 to 3.6, p < 0.001)). Similarly, staff who worked closely with the Knowledge Broker showed significant improvement in the frequency of EIDM-related behaviours (OR 1.33, 95% CI 1.04 to 1.78, p = 0.02). Those not intensively involved, but who sought information from a peer considered an "expert", showed statistically significant improvements in EIDM behaviours (standardized beta coefficient: 0.29, p < 0.0001). Staff who were more central within the social network also showed greater improvement in EIDM behaviour at follow-up (standardized beta coefficient: 0.21, p= 0.09). These positive effects were sustained when organizational mechanisms were present.

## Conclusions

EIDM knowledge, skills and behaviours improved as a result of KTE strategies tailored to the unique needs of each health department. These findings suggest effective methods for developing capacity for EIDM and provide specific support for a tailored approach.

This research was supported by the Canadian Institutes of Health Research (FRN 101867, 126353).

